# Fluorescent Particles Based on Aggregation-Induced Emission for Optical Diagnostics of the Central Nervous System

**DOI:** 10.34133/research.0564

**Published:** 2025-01-24

**Authors:** Shan Liu, Jinkuan Liu, Xue Li, Xiaoxin Du, Cheng Yin, Yong Luo, Chenzhong Li

**Affiliations:** ^1^ Sichuan Provincial Key Laboratory for Human Disease Gene Study, Department of Medical Genetics, Sichuan Academy of Medical Sciences & Sichuan Provincial People’s Hospital, University of Electronic Science and Technology of China, Chengdu 610072, China.; ^2^ School of Medicine, University of Electronic Science and Technology of China, Chengdu 610054, China.; ^3^ Juxintang (Chengdu) Biotechnology Co. Ltd., Chengdu 641400, China.; ^4^ Office of Scientific Research & Development, University of Electronic Science and Technology of China, Chengdu 610054, China.; ^5^ Department of Neurosurgery, Sichuan Provincial People’s Hospital, University of Electronic Science and Technology of China, Chengdu 610072, China.; ^6^ Department of Traditional Chinese Medicine, Sichuan Provincial People’s Hospital, University of Electronic Science and Technology of China, Chengdu 610031, China.; ^7^ Biomedical Engineering, School of Medicine, The Chinese University of Hong Kong, Shenzhen 518172, China.

## Abstract

In 2001, Tang’s team discovered a unique type of luminogens with substantial enhanced fluorescence upon aggregation and introduced the concept of “aggregation-induced emission (AIE)”. Unlike conventional fluorescent materials, AIE luminogens (AIEgens) emit weak or no fluorescence in solution but become highly fluorescent in aggregated or solid states, due to a mechanism known as restriction of intramolecular motions (RIM). Initially considered a purely inorganic chemical phenomenon, AIE was later applied in biomedicine to improve the sensitivity of immunoassays. Subsequently, AIE has been extensively explored in various biomedical applications, especially in cell imaging. Early studies achieved nonspecific cell imaging using nontargeted AIEgens, and later, specific cellular imaging was realized through the design of targeted AIEgens. These advancements have enabled the visualization of various biomacromolecules and intracellular organelles, providing valuable insights into cellular microenvironments and statuses. Neurological disorders affect over 3 billion people worldwide, highlighting the urgent need for advanced diagnostic and therapeutic tools. AIEgens offer promising opportunities for imaging the central nervous system (CNS), including nerve cells, neural tissues, and blood vessels. This review focuses on the application of AIEgens in CNS imaging, exploring their roles in the diagnosis of various neurological diseases. We will discuss the evolution and conclude with an outlook on the future challenges and opportunities for AIEgens in clinical diagnostics and therapeutics of CNS disorders.

## Introduction

The invention of the microscope marked the beginning of biological imaging, unveiling a previously unknown biological world and laying the groundwork for cell theory [1]. Since then, biological imaging has become the first step in human efforts to identify, detect, and regulate biological health and diseases. Today, modern optical diagnostic techniques have made tremendous advancements, such as computed tomography (CT), magnetic resonance imaging (MRI), positron emission tomography (PET), and ultrasound imaging [2]. While these technologies have greatly improved our understanding of diseases and enhanced medical diagnostics and treatment, they also have notable limitations. For instance, CT and PET scans expose humans to ionizing radiation, increasing the risk of cancer. Imaging techniques like MRI are costly, and the spatial resolution and tissue penetration of ultrasound imaging fall short compared to methods like CT and MRI [3].

With the rapid development of high-resolution optical instruments, fluorescence technology has been widely applied in optical diagnostic imaging in recent years [4]. Compared to these optical diagnostic techniques mentioned earlier, fluorescent probes offer advantages such as high sensitivity, high selectivity, noninvasiveness, low toxicity, real-time in situ signal detection, and ease of application, making them highly promising for fields such as quantitative in vitro detection, trace in vivo detection, and early diagnosis and treatment of diseases [5–8].

Although the application of fluorescent probes in optical diagnostic imaging holds great potential, traditional fluorophores face an unavoidable drawback: their fluorescence is typically quenched at high concentrations or in aggregated states, a phenomenon known as concentration quenching or aggregation-caused quenching (ACQ) [9]. The ACQ effect significantly limits the labeling degree of fluorophores to biological analytes, forcing researchers to use diluted probe solutions, which in turn greatly reduces sensitivity and poses a significant barrier to trace-level bioimaging [10]. Fortunately, in 2001, B. Z. Tang serendipitously discovered the phenomenon of aggregation-induced emission (AIE), which exhibits characteristics completely opposite to ACQ in concentrated states [11]. The mechanism behind AIE is restriction of intramolecular motion (RIM), including restriction of intramolecular rotation (RIR) and restriction of intramolecular vibration (RIV) [12]. These compounds do not emit light when dissolved, but they exhibit intense fluorescence in their aggregated state, offering new opportunities for optical diagnostics [11]. We refer to these fluorescent probes with AIE characteristics as AIE luminogens (AIEgens). AIEgens not only inherit the advantages of traditional fluorescent probes but also offer superior penetration depth, enhanced photostability, and higher emission intensity. These characteristics make them highly promising for biomedical research in complex environments, garnering considerable attention from researchers in the field [13,14]. Since 2017, research on AIEgen-based materials has entered a “rapid development phase”, experiencing explosive growth. Data show that since 2016, the annual publication rate of near-infrared (NIR)–AIE research has been steadily increasing, reaching a peak of 106 papers in 2021. The research primarily focuses on 2 main areas: the development of probes and the fine-tuning of their luminescent properties, as well as applications in the biomedical field [15].

The central nervous system (CNS) is one of the most intricate and complex systems in the human body. A significant new study published in *The Lancet Neurology* reveals that in 2021, more than 3 billion people worldwide were affected by neurological disorders, impacting over one-third of the global population and making these disorders a leading cause of illness and disability [16]. Common neurological diseases such as cerebrovascular diseases [17], neurodegenerative diseases [18], and brain tumors [19] have long plagued humanity and still require prompt, accurate diagnosis and treatment [20,21]. As mentioned earlier, many imaging modalities have inherent limitations that are difficult to overcome. Furthermore, due to the unique nature of the CNS, when imaging the brain through intact scalp and skull coverage, spatial and temporal resolution can be significantly affected [22,23]. The distinct advantages of AIEgens, especially their deep tissue penetration, low autofluorescence, low scattering, and high signal-to-noise ratio (SNR), can help improve spatial and temporal resolution, making them suitable for brain imaging. Therefore, AIEgens are emerging as an effective tool for the optical diagnosis of neurological diseases and are promising candidates for use as optical imaging agents [24].

In this review, we summarize and review the AIEgens used in optical diagnostic techniques for various neurological diseases based on the perspectives of clinical needs, such as cerebrovascular disease, neurodegenerative disease, and brain tumors. We also provide new insights into the development of AIEgens in optical diagnostics for CNS diseases, suggesting that AIEgens hold great potential for preclinical/clinical translation in the optical diagnosis of CNS diseases.

## Neurodegenerative Disease

In an aging society, neurodegenerative diseases have become a significant burden affecting nearly every family. Neurodegenerative diseases refer to a series of disorders that progressively damage and destroy the nervous system over time. These diseases typically develop slowly, with their effects and symptoms often appearing later in life. Common neurodegenerative diseases include conditions such as Alzheimer’s disease and Parkinson’s disease [25].

Amyloid aggregation is a prevalent pathogenic alteration in neurodegenerative illnesses [26]. It leads to the formation of amyloid fibrils and plaques, which consistently damage neurons in the brain and impair normal brain function. Using AIEgen imaging technology, we can understand and monitor the formation, metabolism, and even dynamics of amyloid protein aggregation. This is crucial for elucidating the pathogenic mechanisms of amyloid proteins, monitoring diseases with AIEgens, and developing amyloid protein inhibitors [27].

**Fig. 1.**
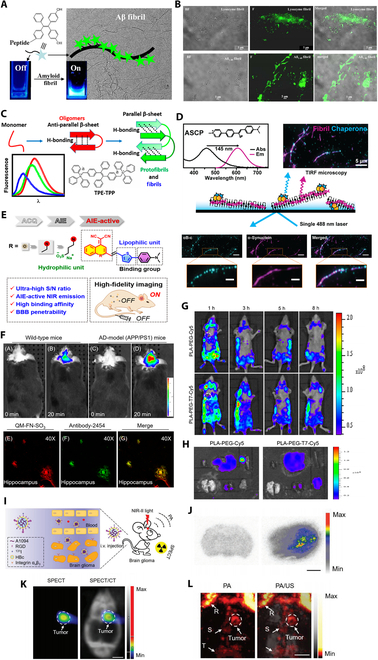
AIE imaging in neurodegenerative disease and CNS tumors. (A and B) “Switch-on” fluorescence probe. (A) The peptide component of the probe offers binding with amyloid fibrils, and this event induces aggregation between TPE components that “switch-on” their fluorescence. (B) Imaging of TPE-peptide-labeled lysozyme fibrils and Aβ_1−40_ fibrils from the same area under bright field (BF) and fluorescence (F). The merged image shows that fluorescence is observed from fibrils only, suggesting that the TPE-peptide binds on fibrils to “switch-on” fluorescence. (C) TPE-TPP fluorescence data identify 3 distinct Aβ aggregation intermediates. (D) ASCP targets and monitors amyloid fibrils, exhibiting red-shifted emission with a large Stokes shift. A representative TIRF microscopy image of αB-c (left, cyan), αSyn stained with ASCP (middle, magenta), and a merged composite (right) is shown. Scale bars, 5 or 2 μm (magnified inset). (E and F) QM-FN-SO3 probes. (E) Probe QM-FN-SO3 structure and its imaging advantages. (F) In vivo and Ex vivo mapping of Aβ deposition in AD model (APP/PS1 transgenic) mice after injection of probe QM-FN-SO3. (G and H) PLA-PEG-T7/TPE self-assembled polymer micelles. (G) In vivo fluorescence images of LN229 tumor-bearing nude mice at different time points. (H) Ex vivo fluorescence imaging of various organs and tumors of LN229 tumor-bearing mice after injection. (I to L) A1094@RGD-HBc probes. (I) Application for accurate mapping of orthotopic brain gliomas. (J) Autoradiography images of the control group (left) and tumor-bearing group (right) after injection of 131I-A1094@RGD-HBc. Scale bar, 2 mm. (K) Micro-single-photon emission computed tomography (MicroSPECT)/CT images and (L) PA/US images of the brain of U87MG tumor-bearing mice at 2 h after injection of 131I radiolabeled A1094@RGD-HBc. R, rostral rhinal vein; S, sagittal sinus; T, transverse sinus.

The aggregation of Aβ fibrils is an instance of amyloid aggregation, with substantial experimental evidence indicating a close association between Alzheimer’s disease and the aggregation of Aβ fibrils in the brain [28]. In the 2015 study by Pradhan et al. [29], they synthesized a “switch-on” AIEgen for the reliable detection and monitoring of amyloid protein fibrils. This probe exhibits no fluorescence in the presence of monomeric proteins/peptides associated with amyloid formation, but it undergoes a fluorescence “turn-on” upon binding to amyloid fibrils (Fig. 1A and B). This probe is composed of 2 main parts: one based on tetraphenylethylene (TPE), which induces green fluorescence through AIE. The other part specifically binds to amyloid proteins and includes the KLVFF component, along with solubilizing segments (RG−/−GR) at both ends. Previous studies indicate that KLVFF represents the core region of amyloid-β (Aβ) protein, responsible for aggregation through hydrophobic interactions between monomers [30]. When the AIEgen probe binds to amyloid proteins, its solubility changes, leading to AIE. Because of the AIE properties, this AIEgens resolve the fluorescence quenching issues encountered by traditional fluorescent probes at high concentrations or in aggregated states. When detecting amyloid fibrils at the same concentration, the fluorescence signal from the AIEgens is approximately 4 times higher than that of the traditional fluorescent probe thioflavin T.

**Table.** Cerebrovascular disease imaging based on AIEgens

Agents	Largest imaging depth (μm)	Highest resolution (μm)	Excitation (nm)	Collection (nm)	Model	Operation	Target disease	Ref.
BTPEBT dots	424	-	800	515–569	C57BL/6J and B6(Cg)-Tyrc-2J/J mice	Cranial window	Normal cerebral vascular	114
XA1 NPs	1,300	-	808	900–1,300	BALB/c nude mice	Intact skull and scalp	Normal cerebral vascular	115
L1013 NPs	-	33.5	808	1,013–1,400	C57BL/6 mice	Intact skull and scalp	Normal cerebral vascular	116
TTB encapsulated by DSPE-PEG2000	1,500	-	728	1,050–1,350	Healthy adult cynomolgus monkeys	Whole skin layer	Normal cerebral vascular	117
OTPA-BBT dots	870	2.4	793	1,100–1,500+	BALB/c mice	Cranial window	Normal cerebral vascular	118
OTPA-BBT dots	700	5.2	793	1,100–1,500+	Callithrix jacchus	Thinned skull	Normal cerebral vascular	118
DCDPP-2TPA NPs	300	2.4	1,550	650+	BALB/c mice	Intact skull	Normal cerebral vascular	122
DCDPP-2TPA NPs	785	-	1,550	650+	BALB/c mice	Cranial window	Normal cerebral vascular	122
DCBT NPs	1,000	10.4	1,300	640	BALB/c mice	Intact skull (SOC)	Normal cerebral vascular	126
DCBT NPs	1,500	3.7	1,300	640	C57 mouse	Cranial window	Normal cerebral vascular	126
TPETPAFN NPs	-	-	515	670	Wistar rats	Cranial window	Cerebral hemorrhage	128
AIE-Gd dots	-	-	512	668	C57BL/6 mice	-	Cerebral hemorrhage	130
TPAQS-NO2	-	-	560	730	ICR mice	-	Cerebral hemorrhage	133
BTF dots	400	0.95	1,550	650	ICR mice	Intact skull	Cerebral ischemia	134
BTF dots	600	1.8	1,550	650	ICR mice	Cranial window	Cerebral ischemia	134

Moreover, the AIEgens overcome other limitations of conventional fluorescent probes, such as low specificity, poor sensitivity, false-positive reactions. The results indicate that the specifically binding AIEgens developed by the team generate signals 8 times higher for detecting amyloid fibrils compared to nonspecifically binding AIEgens. Unlike thioflavin T, this AIEgen’s “Switch-On” feature allows it to emit fluorescence only after binding to the target, thus avoiding false positive effects. Additionally, in the presence of most quenchers—especially Cu^2+^, Fe^3+^, and Au/Ag nanoparticles (NPs)—the fluorescence of thioflavin T is significantly quenched, while the specifically binding AIE probe remains unaffected. This development of AIEgens presents an effective alternative to traditional thioflavin T probes for detecting and monitoring amyloid fibrillation, resulting in more accurate amyloid imaging and the potential for expansion into in vitro diagnostic applications.

Some studies indicate that early oligomers of Aβ are more neurotoxic to neural cells due to structural differences compared to mature fibrils [31–33]. Fluorescent indicator-based technologies are widely used for real-time monitoring of amyloid protein fiber formation due to their ease of application and high sensitivity [34–38]. However, most fluorescence imaging, as mentioned earlier, can only monitor the overall transition from the soluble state to the aggregated state and are unable to detect early oligomers. Therefore, indicators capable of reporting the early transitional steps in amyloid fiber formation would be highly valuable.

In 2020, Das et al. [39] utilized the AIEgen-“TPE-TPP” developed by Leung et al. to label early oligomers of Aβ. They distinguished small oligomeric species, the intermediate species, and the fibrils based on steady-state fluorescence and lifetime characteristics. These 3 types of Aβ aggregates represent early transient protein aggregations, a capability that traditional fluorescent dye ThT struggles to achieve (Fig. 1C). TPE-TPP offers a simple, reliable, and powerful analytical method for reporting atomic-level structural changes in Aβ aggregation. It might be used to assess the interactions between Aβ intermediates and aggregation inhibitors as part of the development of therapeutic strategies for Alzheimer’s disease.

In the same year, Marzano et al. [40] developed a novel AIEgen called ASCP based on the TPE-TPP fluorescent probe, which has a high affinity for binding to α-synuclein (αSyn) fibrils. Lewy bodies (LBs) and Lewy neurites (LNs) are characteristic intraneuronal inclusions in Parkinson’s disease, where αSyn is present in the form of β-sheet-rich amyloid fibrils [41]. Interestingly, while the fluorescence excitation wavelength of ASCP is similar to that of the traditional fluorescent dye ThT, ASCP features a large Stokes shift (145 nm), resulting in a distinct difference in fluorescence color compared to ThT (Fig. 1D). This is primarily due to the restriction of carbon–carbon bond rotation when the dye binds to amyloid structures, leading to changes in the dye’s molecular conformation and electronic structure [42]. The planar conformation restriction extends the electron π-conjugation bridge (specifically the donor–π–acceptor electronic bridge near the C=C bond) and promotes π–π stacking between phenyl groups in the dye molecules. These changes increase electron transfer and lower the excitation energy [43,44]. Thus, the enhanced Stokes shift of ASCP can be attributed to the addition of methylpyridine and dimethylamine groups to the α-cyanostilbene core of ASCP, which enhances its ability for extended electron π-conjugation compared to ThT. This property can be utilized in total internal reflection fluorescence (TIRF) microscopy experiments, allowing for the simultaneous display of fluorescently labeled proteins and ASCP-stained αSyn fibrils using a single laser. ASCP’s enhanced and unique spectral characteristics make it a promising alternative for in vitro studies of amyloid fibrils and their formation mechanisms.

High-fidelity mapping of Aβ plaques is crucial for the early detection of Alzheimer’s disease [45–49]. However, achieving in vivo detection of Aβ plaques is fraught with challenges. To enable AIEgens to perform in vivo imaging of Aβ plaques, they must cross the blood–brain barrier (BBB) to bind to the Aβ plaques in situ. Furthermore, the presence of the skull and other normal tissues also introduces additional challenges for imaging compared to the in vitro environment.

In 2019, Wei Fu’s team designed an AIE particle, QM-FN-SO_3_, for in vivo imaging of Aβ plaques [50] (Fig. 1E). Traditional fluorescent probes, such as commercial thioflavin derivatives (ThT or ThS), are well-known as the gold standard for in vitro amyloid protein fiber histological staining [51,52]. However, they possess inherent drawbacks, including fluorescence signal distortion due to the ACQ effect, persistent background noise caused by ongoing fluorescence, and limited penetration of the BBB, which hinders their application in in vivo imaging [53–55].

To achieve the goal of in situ detection, researchers adopted “Step-by-Step” strategy. First, to address the issues of probe penetration through the BBB and low fluorescence penetration, lipophilic π-conjugated thiophene groups were introduced to enhance the ability of the particles to cross the BBB and adjust their fluorescence wavelength for improved signal penetration. The second step involved replacing ACQ building blocks with AIE building blocks to avoid fluorescence quenching caused by the ACQ effect. Third, to mitigate background noise from persistent fluorescence, the substitution position of the sulfonate group was altered, resulting in QM-FN-SO3 being in a nonfluorescent state when not bound to Aβ plaques. Fluorescence would only become apparent after QM-FN-SO3 binds to Aβ plaques, thereby reducing background noise.

Fluorescence imaging showed that 20 min after intravenous injection of QM-FN-SO3 in mice, the fluorescence intensity in the brain regions of AD model mice was significantly higher than that in wild-type mice (Fig. 1F). This indicates that the QM-FN-SO3 probe successfully penetrated the BBB, specifically captured Aβ plaques in vivo, and achieved high-fidelity mapping of in situ Aβ plaques.

In 2021, Yipu Wang and colleagues designed a novel AIE particle, AIE-CNPy-AD, by analyzing the structure and performance of previously reported small-molecule fluorescent probes for detecting Aβ deposits [56]. The prepared probe, AIE-CNPy-AD, enables high SNR detection of Aβ fibrils in vivo and high-sensitivity, high-fidelity, high-contrast in situ localization of Aβ deposits in vivo. In the brains of 4-month-old AD transgenic mice, this probe could even detect small and sparsely distributed Aβ deposits, a finding that had never been reported in previous AD research.

The researches by Fu and Wang’s team not only aid in the rational design of high-performance AIEgens for accurate in vivo imaging of Aβ deposits but also hold promise as an ideal alternative for early AD diagnosis and reliable monitoring of AD progression. Their studies may also provide a valuable tool for identifying optimal windows for early pharmacological intervention.

MicroRNA-125b (miR-125b) has been shown to be highly expressed in the brain tissue and blood of Alzheimer’s disease (AD) patients, suggesting that it may serve as a promising biomarker for early AD. Its diagnostic results could inform early treatment decisions to prevent severe neuropathological changes associated with AD [57,58].

In 2023, Qin Zhang and colleagues developed a dual “turn-on” biosensor based on the AIE effect and Förster resonance energy transfer (FRET) effect for the in situ detection of the early AD biomarker miR-125b [59]. When miR-125b is present, it binds to the sensor, turning off the FRET effect and restoring the AIEgen signal. The aggregation of AIEgens leads to strong fluorescence emission. Compared to other FRET sensors that do not utilize AIEgens, this biosensor exhibits dual fluorescence enhancement, improving sensitivity.

Experimental results demonstrated that the nano-composite biosensor displayed rapid (≤1 h) and sensitive (<100 pM) biosensing performance for miR-125b in buffer solutions from okadaic acid (OA)-induced AD model mice or in PC12 cells and brain tissues. In vivo imaging experiments revealed a significant increase in fluorescence intensity in the lateral ventricles of AD mice 0.5 h after sensor injection, with the signal reaching its maximum 4 h after injection while also exhibiting excellent spatial resolution. This research not only confirms the feasibility of using FRET and AIEgen technologies as a promising nanoscale platform for in situ and real-time monitoring of AD-related biomarkers but also provides mechanistic insights for the early prognosis of AD.

In summary, AIEgens used in optical diagnostic techniques for neurodegenerative diseases address the fluorescence quenching caused by aggregation-induced ACQ effects that are prevalent in traditional fluorescent probes. They also exhibit strong resistance to fluorescence quenchers, providing brighter fluorescence, low autofluorescence interference, high SNR, and high contrast.

## CNS Tumors

CNS tumors are characterized by the abnormal growth of cells within the brain or spinal cord tissues. [60] The term “CNS tumor” is a broad category encompassing over 120 different types of tumors, each with distinct characteristics and biological behaviors [61].

Among them, malignant brain tumors are the most common destructive diseases affecting the brain. Their aggressive growth within the CNS can quickly lead to noticeable neurological symptoms, resulting in poor prognosis and posing a serious threat to human health [62].

The most common primary malignant brain tumor within the CNS is glioblastoma, which has a high mortality rate [63,64]. Chemotherapy remains one of the primary treatments for gliomas. Recently, targeted drug delivery systems for chemotherapy have been increasingly developed, enhancing the anticancer efficacy of these drugs and reducing side effects on normal cells [65–67]. However, most drug delivery systems can deliver drugs to cancer cells but cannot monitor their distribution and release within these cells [68,69].

In 2021, Hu et al. [70] developed a fluorescence-based delivery system, PLA-PEG-T7, using AIE dyes. First, the AIE-based fluorescent delivery system addresses the fluorescence quenching caused by the ACQ effect in traditional fluorescent molecules, providing strong fluorescence and excellent cell imaging capabilities. Second, to enhance the drug delivery system’s ability to cross the BBB and blood–tumor barrier (BTB), Hu et al. used peptide T7, which targets the transferrin receptor (TfR) that is overexpressed in brain capillary endothelial cells and many malignant tumor cells, and is significantly more present than in normal cells. This imparts the PLA-PEG-T7 system with exceptional BBB and BTB penetration capabilities. Third, they utilized polylactic acid as the framework for the fluorescent drug delivery system, offering the benefits of easy biodegradability, nontoxic degradation products, and metabolism-based elimination. Our focus is primarily on the bioimaging capabilities of this system. Experimental results indicate that, compared to nontargeted fluorescent drug delivery systems, the T7-targeted modification of this fluorescent delivery system yields significant fluorescence signals in the tumor region within 1 h after intravenous injection into LN229 (glioma cell line) tumor-bearing mice. The fluorescence intensity in the tumor area markedly increases after 3 h, demonstrating its capability to cross the BBB and BTB, precisely targeting the tumor site and enabling in vivo bioimaging via AIE fluorescence effects (Fig. 1G and H). Furthermore, additional experiments have shown that this system has excellent tumor-targeting properties, effective anti-tumor activity when delivering chemotherapeutic drugs, nontoxicity in the absence of drugs, and good blood compatibility. These attributes make it a promising drug delivery vehicle for comprehensive cancer therapy, capable of both targeted disease assessment and treatment.

Another notable study on glioma imaging and treatment is a 2022 study by Su et al. [71], where they developed a novel biomimetic nanocomposite, TMPsM, designed to enable tumor-targeted imaging and precision therapy based on the AIE effect. In their approach, transglutaminase 2 (TG2) was utilized as an activator, given its overexpression in glioblastoma multiforme (GBM) [72] and its association with cancer progression [73]. To enhance the biocompatibility, BBB and BTB penetration, and retention time of TMPsM, they coated it with TfR aptamer-modified B16F10 cell membranes. Upon crossing both barriers into the tumor microenvironment (TME), TMPsM disassembles to release AIE particles and apoptosis-inducing small interfering RNA (siRNA). AIE particles bind with TG2, inducing fluorescence due to increased aggregation, enabling high SNR AIE-based fluorescence imaging for precise tumor localization and dynamic TG2 activity monitoring, facilitating accurate GBM diagnosis. Meanwhile, siRNA silences TG2, triggering tumor cell apoptosis for targeted therapeutic effects. In vivo results showed that in GBM (U87MG)-bearing mice, significant brain accumulation was observed 2 h after injection of TMPsM, accurately localizing glioma cells. Notably, fluorescence signals persisted for at least 24 h, indicating successful BBB and BTB penetration, precise glioma targeting, and effective in vivo imaging capabilities.

Besides chemotherapy, another treatment method for gliomas is surgical resection. Positive surgical resection following imaging examination is the standard clinical treatment [74]. However, the inability to accurately assess tumor location and the unclear boundaries of intracranial surgeries limit the diagnosis and treatment of brain gliomas [75].

In previous studies, copper sulfide has often been used as an optical imaging agent for in vivo glioma localization [76]. However, due to the brain tissue’s higher scattering and absorption properties, higher doses of copper sulfide are required, which may lead to severe adverse reactions and toxicity [77]. Additionally, there are significant challenges related to crossing the BBB [78–81]. AIEgens hold broad application potential for in situ imaging of deep-seated brain tumors. By delineating the margins of gliomas, AIEgens assist in accurate imaging and image-guided surgery.

In 2018, Sheng et al. [82] synthesized a novel AIEgen-based bioprobe for glioma imaging. They covalently attached the tumor-specific c-RGD peptide, which targets αVβ3 integrin receptors overexpressed in gliomas [83–85], to the synthesized AIEgens, creating TB1-RGD dots for targeted glioma imaging. Experimental results showed that after injecting TB1-RGD into brain tumor model mice, the tumor signal intensity was significantly higher at all time points compared to TB1 alone, with maximum intensity reached at 24 h after injection and clear imaging depth up to 2.0 mm. This demonstrated the TB1-RGD dots’ capability for accurate glioma targeting, overcoming skull and surrounding tissue interference to achieve effective in vivo glioma imaging and localization.

The following year, Liu et al. [86] advanced Sheng’s work by developing a novel π–π conjugated ionic organic molecule, A1094, for glioma imaging. This probe leveraged the aggregation-induced absorption enhancement (AIAE) effect—a photophysical phenomenon where, in addition to fluorescence enhancement, absorption efficiency significantly increases upon aggregation (Fig. 1I). This effect not only boosted imaging signal intensity but also improved imaging depth. To enhance the targeting specificity and BBB penetration of A1094, the molecule was encapsulated in an RGD-modified hepatitis B virus core protein (RGD-HBc), forming A1094@RGD-HBc. In vivo imaging results showed that low doses of A1094@RGD-HBc injected into U87MG-bearing mice enabled imaging of tumors at a depth of 5.9 mm beneath the scalp—1.47 times deeper than previously reported results [87]. Notably, the AIAE effect allowed A1094@RGD-HBc to achieve this depth with only a quarter of the dose required by the traditional ICG@RGD-HBc imaging agent [88]. Further in vivo imaging confirmed A1094@RGD-HBc’s outstanding targeting specificity, imaging accuracy, and biocompatibility, while it exhibited superior photostability, specific tumor targeting, and efficient, concentration-independent absorption compared to inorganic absorbers like gold nanostars [89] (Fig. 1J to L).

Fluorescent probes based on AIE and AIAE for glioma imaging not only surpass conventional probes but also have shown potential to combine in situ fluorescence imaging with precision-targeted therapy. This innovation allows not only glioma diagnosis but also directed monitoring and evaluation of chemotherapy efficacy, as well as more precise localization for surgical intervention on glioma cells. With these advantages, AIE and AIAE probes are anticipated to have vast applications in deep-seated, in situ brain tumor imaging and are poised for clinical translation.

## Demyelinating Disease

Demyelinating diseases are conditions that cause damage to the myelin sheath in the brain, spinal cord, and nerves. When myelin is affected, it alters the way nerves communicate and function, leading to corresponding clinical symptoms such as blurred vision, tingling, or numbness in body parts. Common demyelinating diseases include multiple sclerosis and Guillain–Barré syndrome [90–94]. The prevalence of demyelinating diseases is significant; for instance, a 2019 study estimated that nearly 1 million people in the United States are affected by multiple sclerosis alone [95].

Visualizing myelin can greatly enhance the diagnosis of myelin-related diseases and improve our understanding of brain function [96]. Fluorescence-based myelin imaging can target and observe the distribution of areas of interest with higher spatial resolution compared to common unlabeled imaging techniques [97]. However, some common myelin-specific fluorescent probes, such as the commercial probe Vybrant DiD (DiD), require complex tissue preprocessing to achieve good tissue permeability for deep tissue myelin imaging. This preprocessing can damage some fine structures of the myelin, significantly reducing the selectivity of myelin staining [98].

**Fig. 2.**
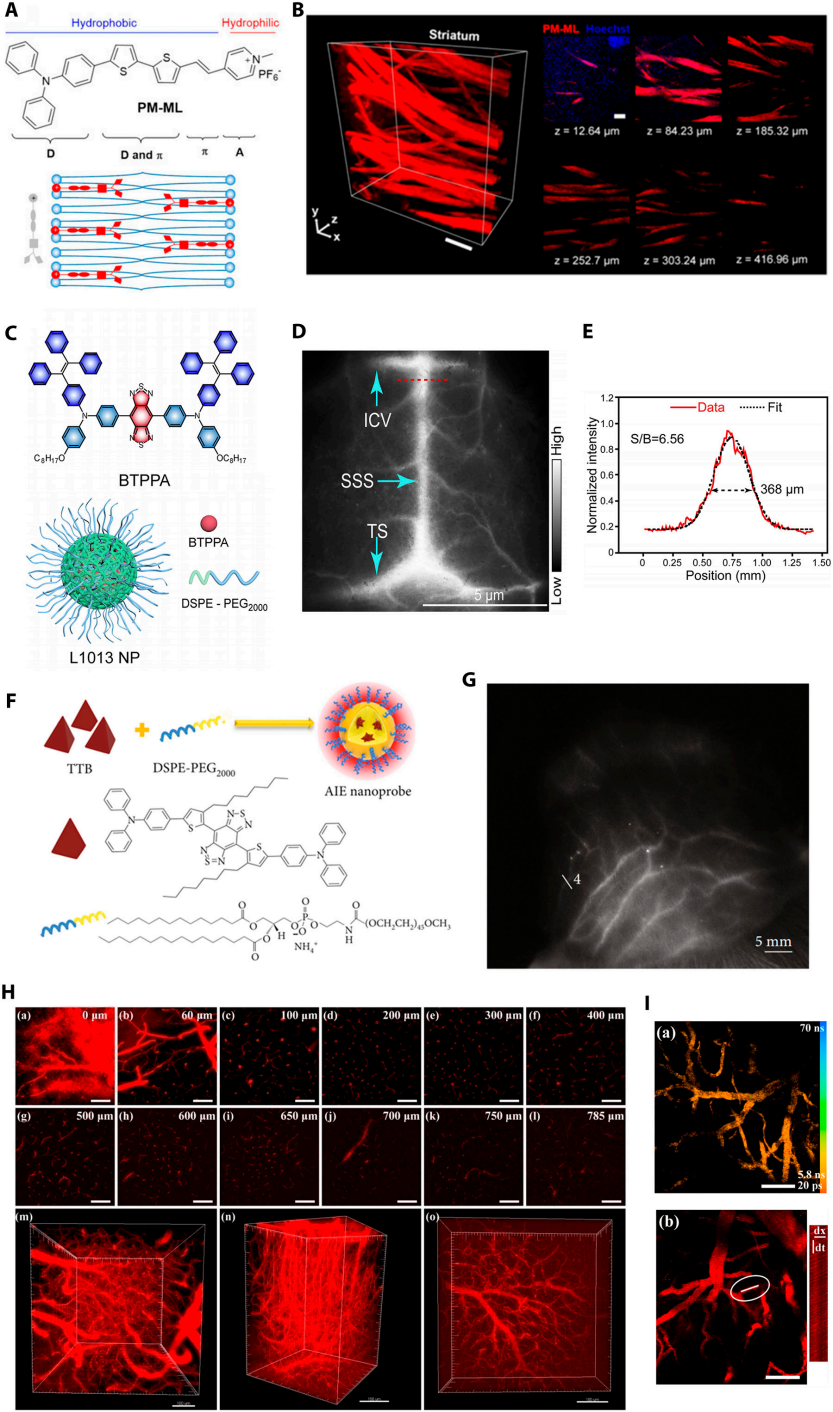
AIE imaging in demyelinating disease and cerebrovascular disease. (A and B) PM-ML for high-performance myelin imaging. (A) Chemical structure, schematic illustration of PM-ML structural characteristics, and its interaction with membrane structure. (B) 3D rendered Z-stack of the STR in the ClearT mouse brain labeled with PM-ML. (C to E) Organic NPs (L1013 NPs) were synthesized for in vivo imaging. (C) Chemical structure of BTPPA molecule and the schematic illustration of L1013 NP. (D) Transcranial fluorescence images of cerebral vessels 2 min following intravenous injection of L1013 NPs. (E) Cross-sectional intensity (red solid line) and Gaussian fit fluorescence intensity profiles (black dotted line) with L1013 NPs imaging. (F and G) NIR-II AIE probes. (F) Schematic illustrations of preparation of AIE probes through a nanoprecipitation method. (G) NIR-II fluorescence imaging of the scalp vasculature in the cynomolgus monkey after intravenous injection of the AIE probes. (H and I) DCDPP-2TPA probes. (H) In vivo 3PFM images of the brain blood vessels on a mouse with skull opened. (I) In vivo functional imaging of the brain blood vessels on a mouse with intact skull.

To address the aforementioned issues, in 2021, Chen’s team reported an AIE-active probe called PM-ML (Fig. 2A), which exhibited significant fluorescence enhancement upon interaction with myelin [99]. PM-ML targets the lipids rich in myelin [100,101] to achieve specific localization of myelin. Compared to the commercial myelin probe DiD, PM-ML demonstrates excellent photostability while specifically labeling cell membranes in both live and fixed cells, enabling selective staining of myelin regions in mouse brains with a high signal-to-background ratio (SBR). Because of the high labeling specificity and high SBR of PM-ML, researchers further explored its application for 3-dimensional (3D) visualization of myelinated fiber bundles in thick brain sections. The results showed that PM-ML successfully achieved 3D rendering of the striatum (STR) from the ClearT mouse brain (Fig. 2B). Additionally, they used PM-ML to experimentally validate a demyelinating disease model, demonstrating the lack of myelin formation in a low myelin tremor mutant mouse model of multiple sclerosis, thus confirming the utility of PM-ML staining in demyelinating disease research.

In summary, fluorescence probes like PM-ML based on AIEgens can serve as multifunctional and reliable tools for visualizing myelinated fibers in the CNS and peripheral nervous system (PNS). They provide researchers with clearer images with high SNRs, allowing for better analysis of myelin density, bundle structures, fiber trajectories, and detailed morphology of individual myelinated fibers. This can further reveal the subtle remodeling of myelin during development, disease progression, or therapeutic responses.

## Cerebrovascular Disease

The structure of healthy brain vasculature maintains the brain’s internal environment, but when this structure and function undergo dysregulated changes, cerebrovascular diseases can occur. Cerebrovascular diseases, which impact blood flow to the brain, include conditions such as stroke, cerebral hemorrhage, and carotid atherosclerosis. Overall, cerebrovascular diseases primarily encompass 2 pathophysiological processes: cerebral ischemia and cerebral hemorrhage. These conditions can restrict cerebral blood supply, potentially damaging brain structures and impairing normal brain function [102].

The ability to visualize brain vasculature in vivo is essential for understanding cerebral biological processes, including angiogenesis, vascular leakage, and leukocyte extravasation [103,104]. Current probe materials for in vivo vascular imaging, such as organic dye-labeled biopolymers [104,105] and inorganic semiconductor quantum dots (QDs) [106], have significant limitations. For example, they often exhibit weak signal output, high biotoxicity, and small molecular probes prone to vascular leakage [107]. To address these issues, fluorescent probes have been developed; however, they introduced new challenges, such as fluorescence quenching caused by the ACQ effect, hindering advancements in vascular imaging [108–110].

Fluorescent probes with AIE properties have addressed these 2 primary issues. By avoiding the ACQ effect and achieving high signal output, AIE-based probes hold a substantial promise for applications in brain vascular imaging.

Additionally, reducing spontaneous interference, enhancing tissue penetration, and achieving higher resolution and SNR are essential for noninvasive imaging of deep brain microvessels in vivo. The NIR biological window, particularly the second NIR (NIR-II) window (1,000 to 1,700 nm), presents unique advantages, including ultra-low photon scattering and improved tissue penetration, making it an increasingly popular choice for AIEgen-based research applications [111,112].

For example, by extending π-conjugation and introducing robust donor–acceptor (D-A) systems, AIEgens can be modified to achieve significant red-shifted emission, allowing them to reach the NIR-II window [82,113]. Many high-quantum-yield NIR-II luminogens have been developed, demonstrating outstanding performance in cerebral vascular imaging applications.

In this section, we will outline specific examples of AIEgens applied to cerebral vascular imaging, including many that utilize NIR-II for enhanced tissue penetration. We will start with imaging normal cerebral blood vessels and then focus on the 2 pathophysiological processes of cerebrovascular diseases—cerebral ischemia and cerebral hemorrhage—examining how AIEgens utilize their various advantages to effectively image and diagnose these conditions.

### Normal cerebral vascular

In 2013, Liu and Tang conducted the first study using AIEgen NPs for in vivo cerebral vascular imaging. They synthesized a novel AIEgen, 4,7-bis[4-(1,2,2-triphenylvinyl)phenyl]benzo-2,1,3-thiadiazole (BTPEBT) [114]. Experimental results showed that its average brightness surpassed that of the commercial vascular imaging probe QD655. Their study also demonstrated that BTPEBT achieved an imaging depth of 424 μm in brain tissue vessels with the mouse skull removed, producing clear 3D images. When the mouse skull was retained, the imaging depth reached 132 μm beneath the skull in brain tissue vessels. This research confirmed that AIEgens possess excellent colloidal stability, superior photostability, and low toxicity in vivo, marking the beginning of a new era in the use of AIEgens for vascular imaging. Since then, more AIEgen probes have been developed specifically for vascular imaging applications.

In subsequent studies, the imaging depth of AIE fluorescence has been further improved. In the Xu et al. [115] research, leveraging the powerful imaging capabilities of NIR-II, a new D-A-D type NIR-II emitting AIE molecule called XA1 was developed by extending π-conjugation and introducing strong D-A systems. With the mouse scalp and skull intact, the imaging depth of AIE fluorescence in cerebral vessels increased to 1,300 μm beneath the skin. In the Wu et al. study [116], they overcame the challenge of low fluorescence yield by synthesizing AIE NPs (L1013 NPs) with a high fluorescence quantum yield of 9.9% (Fig. 2C). When applying L1013 NPs, the highest spatial resolution for NIR imaging of cerebral vessels through the intact skull and scalp reached 33.5 μm. During brain vascular imaging in mice, the researchers were able to clearly image cerebral vessels, such as the transverse sinus, superior sagittal sinus, and inferior cerebral veins, through the intact scalp and skull using low power density and short exposure times (Fig. 2D and E). Notably, earlier AIE imaging was primarily based on a cranial window, but it has since progressed to imaging through the scalp and skull. This advancement holds significant importance for future clinical applications in human imaging.

In Sheng et al. study [117], AIE dots were used for the first time in cerebral vascular imaging of nonhuman primates, marking a significant advancement in the clinical translation from rodents to primates (Fig. 2F). By using bright and nontoxic AIE probes for fluorescence imaging in the second NIR window (NIR-II, 1,000 to 1,700 nm), they overcame the limitations of existing millimeter-depth NIR-II fluorescence imaging, achieving unprecedented 1.5-cm-deep vascular imaging in nonhuman primates (Fig. 2G).

In Feng et al. study, they designed and synthesized a NIR-II AIEgen, OTPA-BBT dots, which can be excreted via the hepatobiliary and gastrointestinal pathways and used for brain vascular imaging. After being encapsulated into biological NPs with F127, OTPA-BBT dots exhibited an emission peak at 1,020 nm and an exceptionally high quantum yield of 13.6%. Additionally, they emitted ultrabright fluorescence beyond 1,100 nm and even up to 1,500 nm in the NIR-IIb range. High-resolution cerebrovascular imaging through the thinned skull of marmosets allowed for the clear identification of 5.2-μm capillaries at a depth of 200 μm, with a visualization depth reaching approximately 700 μm below the thinned skull. Moreover, OTPA-BBT dots could potentially be used for high temporal resolution functional scans through the marmosets’ thinned skulls to track cerebral blood flow. Real-time visualization revealed the occlusion of cortical vasculatures underneath the thinned skull, allowing for the observation of blood flow halt or even reflux in other side branches [118].

The NIR window—especially the NIR-II window—enables greater tissue penetration depth, facilitating in vivo AIEgen imaging of cerebral vasculature. Similarly, multiphoton imaging technology, with its unique multiphoton characteristics, ensures high penetration depth while achieving precise “point” localization. Among these, 3-photon imaging technology is one of the most extensively studied multiphoton imaging techniques. Compared to confocal and 2-photon excitation microscopy, 3-photon imaging significantly enhances the SBR, utilizes longer excitation wavelengths, and has higher nonlinear fluorescence constraints, demonstrating the potential for deep imaging beyond 1 mm [119–121]. The application of 3-photon imaging technology has led to the synthesis of AIEgens for 3-photon imaging. These AIEgens can image through an intact skull without invasive procedures, thereby reducing the organism’s pain and injury.

A deep-red emissive AIEgen, DCDPP-2TPA, was synthesized and encapsulated in Pluronic F-127 to create NPs for 3-photon fluorescence imaging of mouse brain arteries, enabling brain vasculature imaging without the need for craniotomy or skull-thinning procedures [122]. The 3-photon absorption cross-section of DCDPP-2TPA NPs is 2.95 × 10^−79^ cm^6^ s^2^, which is larger than previously reported organic dyes. Additionally, due to the minimal decrease in 3-photon fluorescence intensity, the NPs exhibit excellent photostability even after prolonged continuous scanning. In in vivo imaging of mouse brain vasculature, DCDPP-2TPA NPs demonstrated a high SNR in detecting arteries at depths of up to 785 μm and distinguishing capillaries as small as 2.4 μm at a depth of 300 μm (Fig. 2H and I).

Another NIR-II-based AIE fluorescent imaging study has, for the first time, achieved dual-channel deep imaging of brain vasculature and neurons beneath an intact skull. In 2022, He et al. [123] employed a strategy combining skull optical clearing (SOC) technology with 3-photon fluorescence microscopy (3PM) to mitigate the high scattering effects caused by the skull and brain tissue. This approach enabled deep AIE imaging of brain vasculature and tissue.

The team utilized visible–NIR-II-compatible skull optical clearing agents (VNSOCA), introducing D_2_O solutions to replace H_2_O, which achieved optical clearing of the skull by locally applying these chemicals for refractive index matching without requiring invasive craniotomy [124]. This method not only reduced optical scattering caused by the skull but also prevented the environmental changes and unavoidable inflammatory responses associated with craniotomy [125].

Additionally, the researchers synthesized a novel AIEgen, DCBT NPs, characterized by strong D-A features and extended π-conjugation. These properties resulted in NIR emission and significantly higher σ3 values than those of conventional dyes [126]. DCBT NPs exhibited high brightness, remarkable photostability, and excellent biocompatibility, enabling precise imaging. Experimental results showed that DCBT achieved a maximum σ3 value of 3.53 × 10^−78^ cm^6^ s^2^ photon^-2^ within the 1,300-nm range, significantly surpassing previously reported organic probes. Over 16 min of continuous irradiation, the 3PF intensity decreased by only 30%. By combining DCBT NPs with VNSOCA, the researchers achieved an imaging depth of 1.5 mm in craniotomized mice, 1.0 mm for brain vasculature beneath an intact skull, and over 700 μm for neuron imaging depth. This marks the greatest brain vasculature imaging depth achieved using AIEgens beneath an intact mouse skull to date.

### Cerebral hemorrhage

BBB leakage is a pathophysiological process associated with hemorrhagic cerebrovascular diseases [127]. Early detection of BBB leakage is crucial for the early diagnosis of these conditions, as timely intervention can prevent the potentially harmful effects of impaired cerebral perfusion on brain cells caused by hemorrhage.

To accurately monitor the leakage of the BBB at various stages within the CNS, Cai et al. [128] designed TPETPAFN with AIE characteristics. The researchers conducted their studies using a photothrombotic ischemia (PTI) rat model, which can induce ischemic stroke in a single vessel with precise localization, controllable size, and clear boundaries, ensuring accurate assessment of BBB integrity. Compared to the traditional contrast agent Evans Blue (EB) used for assessing BBB damage [129], TPETPAFN NPs exhibit higher sensitivity and lower toxicity. This ensures their potential applicability in both preclinical and clinical settings. Interestingly, the size of NPs is correlated with their brightness. In vivo studies showed that in the PTI model, 60-nm NPs exhibit the highest brightness among all sizes but cannot penetrate the BBB. On the other hand, 10-nm NPs can cross the BBB, but they have lower sensitivity and the weakest fluorescence. The study found that 30-nm NPs are the most effective and specific probes for evaluating BBB damage. This indicates that the diameter of AIEgens is closely related to the accuracy of detecting BBB leakage.

Additionally, another group of researchers used TPETPAFN to create AIE-Gd nanodots by encapsulating it in lipid-PEG (polyethylene glycol) and combining it with gadolinium (Gd) [130]. These nanodots can be used to detect BBB leakage and microvascular hemorrhage. The researchers used experimental cerebral malaria (ECM) mouse models to simulate brain hemorrhage and BBB leakage. The study found that even in the smallest capillaries of the pia mater, the negative control group, i.e., normal mouse arteries, showed no significant increase in interstitial background signal or extensive leakage of AIE-Gd dots from blood vessels. In contrast, in the positive model group, AIE-Gd nanodots formed punctate aggregates and infiltrated the interstitium around hemorrhagic microvessels, a phenomenon not observed with EB. AIE-Gd dots not only feature fluorophores with AIE characteristics as the core, providing bright fluorescence for optical imaging, but also contain Gd, which enables quantitative measurement of vascular leakage and tissue accumulation of AIE-Gd through inductively coupled plasma mass spectrometry (ICP-MS). This allows for precise detection of BBB leakage and microvascular hemorrhage.

### Cerebral ischemia

Cerebrovascular diseases such as vascular stenosis (narrowing), thrombus formation, and embolism can all lead to cerebral ischemia. The common pathological feature of these conditions is hypoxia, caused by arterial blockage and reduced blood supply [131].

In hypoxic microenvironments, nitroreductase (NTR) is consistently overexpressed and is considered a sensor of hypoxia severity [132]. As an activatable probe for hypoxia detection, TPAQS-NO2 was designed to respond to NTR, consisting of an electron-donating triphenylamine group and an electron-accepting quinolinium salt group [133]. When activated, TPAQS-NO2 exhibits a significant Stokes shift (186 nm) and has high specificity for NTR, thereby avoiding interference caused by molecular self-absorption. When TPAQS-NO2 was injected into the true-operated group of ischemic stroke rodent models, a strong fluorescence signal was observed in the brain region within 1 h after injection and continued to intensify over 4 h. In contrast, the sham-operated group showed significantly lower fluorescence signals.

Unlike the indirect diagnosis achieved through biomarker detection, such as NTR detection, AIEgens also enable the direct detection of cerebral thrombosis. Wei Qin and colleagues developed an AIEgen, BTF dots, based on the principle of 3-photon imaging [134]. Unlike 2-photon imaging, which can typically only achieve shallow imaging at the cranial window [135], resulting in unavoidable disturbance to the native environment and brain tissue inflammation [136], 3-photon imaging utilizes high-order nonlinear local excitation in the NIR-II region (1,000 to 1,700 nm). This significantly enhances penetration depth, spatiotemporal resolution, and SBR [137].

These dots leverage the high precision of 3-photon localization and the high penetration capabilities of far-red (FR)/NIR frequencies to achieve an ultimate spatial resolution of 0.95 μm through the entire cranial layer. The D-A structure endows BTF with FR/NIR emission and notable multiphoton absorption capabilities. The presence of more freely rotating benzene rings and t-Bu groups facilitates the AIE process, while the twisted two-photon absorption (TPA) groups and bulky t-Bu groups hinder the formation of strong π–π stacking interactions. These combined factors result in BTF having long-wavelength emission and high quantum efficiency.

Reportedly, BTF dots exhibit efficient emission in the FR/NIR region with an efficiency of 36.1% and a 3-photon absorption cross-section of 2.56 × 10^−79^ cm^6^ s^2^ at 1,550 nm. The corresponding NPs demonstrate an emission peak reaching the NIR spectrum at 645 nm and an absorption peak at 500 nm. In vivo examination of mouse brain vasculature with an intact skull revealed that the diameters of the capillaries at depths of 200, 300, and 400 μm were 0.95, 1.59, and 2.08 μm, respectively. Additionally, BTF dots are the first AIE particles that utilize AIEgens to observe the cerebral thrombosis process through an intact skull in a mouse model. This demonstrates their potential for deep-tissue imaging in vivo, particularly for brain imaging, offering excellent penetration and superior image clarity.

Some AIEgens have been developed not only to image pathological phenomena associated with disease but also to study the diseases directly. For instance, Saba et al. [138] developed an AIEgen that also utilizes 3-photon fluorescence imaging, specifically targeting atherosclerosis, a vascular disease affecting both the carotid arteries supplying blood to the brain and the cerebral vascular system itself.

To overcome the limitations of existing lipid-specific luminescent molecules, which are often hindered in vivo due to their high hydrophobicity, Wang’s team [139] designed TPAPhCN, an AIEgen with AIE characteristics suitable for 3-photon fluorescence imaging of lipid-rich tissues, such as fatty liver and atherosclerotic plaques in brain and carotid arteries. TPAPhCN dots remain stable in aqueous solutions and produce high 3-photon fluorescence in the FR/NIR region under NIR-II laser excitation, enabling in vivo lipid labeling and imaging.

In a mouse model, TPAPhCN demonstrated an exceptional imaging depth of 1,000 μm, allowing for 3D 3-photon images of brain arteries with bright fluorescence in lipid-rich plaques. This advancement in fluorescent labeling of lipids, particularly atherosclerotic plaques, highlights the potential of AIEgens in the diagnosis and monitoring of atherosclerosis and in the assessment of arterial plaques.

## Discussion

In recent years, AIEgens have emerged as transformative tools for biological imaging, with specific applications across various neuropathological conditions. Their unique properties—such as resistance to ACQ, high photostability, and suitability for deep-tissue imaging—have made them especially valuable in the field of neuroimaging. Most AIEgen fluorescent probes used in bioimaging are derived from compounds such as TPE and TPA. A common strategy for designing biomolecule-responsive AIEgens is to link these compounds with cleavable chemical bonds or functional groups, which alter the water solubility of AIEgens in specific reactions. These unique modifications enable AIEgens to selectively image biological targets [140]. AIE-based probes are instrumental in advancing the visualization of essential neural structures, including Aβ plaques in Alzheimer’s disease, demyelinated regions in multiple sclerosis, and the complex vascular architecture in cerebrovascular diseases. Such imaging capabilities provide unprecedented insights into both the progression of neural diseases and the efficacy of therapeutic interventions.

For Alzheimer’s disease, the development of AIE-based probes has allowed more selective and detailed visualization of Aβ aggregates, a hallmark of disease pathology. By overcoming the limitations of traditional fluorescent dyes—such as limited specificity and photobleaching—AIEgens like TPE-TPP have enabled a significant improvement in spatial resolution and depth, thus facilitating early and accurate diagnosis. This approach paves the way for monitoring disease progression in vivo, a capability that could revolutionize both research and clinical practices in Alzheimer’s disease management.

In the domain of demyelinating diseases, the introduction of AIEgens, exemplified by the PM-ML probe, has provided powerful imaging tools to observe myelin integrity with high specificity. The ability of PM-ML to selectively bind to myelin lipids has proven beneficial in studying demyelination patterns in animal models of multiple sclerosis. The selective staining capability and high SNR offered by PM-ML support its application in developing more accurate models of disease progression and recovery in response to therapeutic interventions. Furthermore, PM-ML’s use in 3D visualization of myelinated fiber bundles facilitates a deeper understanding of the intricate relationship between structural changes and functional impairment in the CNS.

For cerebrovascular diseases, AIEgens have made possible the high-resolution imaging of brain vasculature under noninvasive conditions. The deployment of NIR and NIR-II AIEgens in vascular imaging has circumvented challenges like light scattering and shallow penetration depth associated with traditional probes. AIEgen-based NIR imaging provides clinicians with powerful, noninvasive imaging modalities, particularly in detecting the BBB disruptions in hemorrhagic and ischemic strokes. Notably, the development of 3-photon imaging using AIEgens has achieved high-resolution imaging through an intact skull, which not only enhances clinical feasibility but also reduces the potential for inflammation and artifact generation during imaging. This capability to visualize cerebral vessels at micrometer-level resolution enables researchers to observe vascular remodeling and pathological changes in real time, advancing our understanding of cerebrovascular pathology and therapeutic response.

While AIEgens have demonstrated a wide range of advantages, several challenges remain for their full integration into clinical applications. First, the biocompatibility and long-term safety of AIEgens require further investigation, especially given the varying properties of different AIEgens in terms of metabolism, excretion, and potential accumulation in tissues. Ensuring that AIEgens are nontoxic and can be safely used over prolonged periods is essential for their application in chronic disease imaging. Second, although recent developments in NIR-II AIEgens have improved imaging depth and resolution, there remains a need to balance these advantages with signal stability and fluorescence intensity under physiological conditions. Achieving optimal SNRs in deep tissues will be crucial for enhancing diagnostic accuracy and sensitivity in clinical settings.

In addition to addressing these challenges, future research should focus on developing multifunctional AIEgens that can simultaneously target multiple biomarkers or disease processes. For example, combining AIEgens with other imaging modalities such as MRI or PET could yield multimodal probes that provide complementary information, enhancing diagnostic accuracy. Similarly, advances in photo-switchable or stimulus-responsive AIEgens could lead to real-time monitoring of dynamic changes in disease states, such as BBB integrity in stroke or Aβ deposition in Alzheimer’s disease. Such probes would not only contribute to early diagnosis but also facilitate real-time monitoring of therapeutic efficacy.

Furthermore, as AIEgen technology progresses, the development of probes tailored to specific neuropathologies will become more feasible, potentially leading to personalized diagnostic and therapeutic strategies. For instance, in conditions like Alzheimer’s and multiple sclerosis, patient-specific AIEgens could be designed to reflect individual variations in biomarker expression and distribution, enabling a more tailored approach to both disease monitoring and treatment response. Additionally, clinical translation of AIE-based imaging requires overcoming regulatory hurdles and establishing standardized protocols to ensure consistent and reproducible results across diverse patient populations and imaging platforms.

In conclusion, AIEgens hold transformative potential in advancing neuroimaging for Alzheimer’s, demyelinating, and cerebrovascular diseases. Their unique characteristics have already set new standards for imaging clarity, specificity, and depth, and continued research and development in this field promise even greater impacts on early diagnosis, therapeutic monitoring, and personalized medicine. By addressing current limitations and exploring innovative applications, AIE-based probes could become indispensable tools for unraveling the complexities of brain disorders and improving outcomes for patients worldwide.
